# Greater Osseointegration Potential with Nanostructured Surfaces on TiZr: Accelerated vs. Real-Time Ageing

**DOI:** 10.3390/ma14071678

**Published:** 2021-03-29

**Authors:** Andreas Stavropoulos, Rebecca Sandgren, Benjamin Bellon, Anton Sculean, Benjamin E. Pippenger

**Affiliations:** 1 Division of Regenerative Dental Medicine and Periodontology, University of Geneva, CH-1211 Genève 4, Switzerland; 2 Department of Periodontology, Faculty of Odontology, Malmö University, SE-205 06 Malmö, Sweden; 3 Division of Conservative Dentistry and Periodontology, University Clinic of Dentistry, Medical University of Vienna, AT-1090 Vienna, Austria; 4 Department of Biomedicine, Medical Faculty, Lunds University, SE-223 62 Lund, Sweden; rebecca.sandgren@med.lu.se; 5 Department of Preclinical & Translational Research, Institut Straumann, CH-4002 Basel, Switzerland; benjamin.bellon@straumann.com; 6 Department of Periodontology, Faculty of Dentistry, University of Zurich, CH-8032 Zurich, Switzerland; 7 Department of Periodontology, School of Dental Medicine, University of Bern, CH-3210 Bern, Switzerland; anton.sculean@zmk.unibe.ch

**Keywords:** osseointegration, hydrophilicity, nanostructured materials, rabbits, Roxolid, SLActive, SLA

## Abstract

Surface chemistry and nanotopography of dental implants can have a substantial impact on osseointegration. The aim of this investigation was to evaluate the effects of surface chemistry and nanotopography on the osseointegration of titanium-zirconium (TiZr; Roxolid^®^) discs, using a biomechanical pull-out model in rabbits. Two discs each were placed in both the right and left tibiae of 16 rabbits. Five groups of sandblasted acid etched (SLA) discs were tested: (1) hydrophobic without nanostructures (dry/micro) (*n* = 13); (2) hydrophobic with nanostructures, accelerated aged (dry/nano/AA) (*n* = 12); (3) hydrophilic without nanostructures (wet/micro) (*n* = 13); (4) hydrophilic with nanostructures, accelerated aged (wet/nano/AA; SLActive^®^) (*n* = 13); (5) hydrophilic with nanostructures, real-time aged (wet/nano/RTA). The animals were sacrificed after four weeks and the biomechanical pull-out force required to remove the discs was evaluated. Adjusted mean pull-out force was greatest for group wet/nano/RTA (64.5 ± 17.7 N) and lowest for group dry/micro (33.8 ± 10.7 N). Multivariate mixed model analysis showed that the pull-out force was significantly greater for all other disc types compared to the dry/micro group. Surface chemistry and topography both had a significant effect on pull-out force (*p* < 0.0001 for both), but the effect of the interaction between chemistry and topography was not significant (*p* = 0.1056). The introduction of nanostructures on the TiZr surface significantly increases osseointegration. The introduction of hydrophilicity to the TiZr implant surface significantly increases the capacity for osseointegration, irrespective of the presence or absence of nanotopography.

## 1. Introduction

A sandblasted, large-grit, acid-etched surface has become one of the most extensively used surface for dental implants. High dental implant survival rates have been demonstrated in several long-term clinical trials [1,2,3]. In recent years, efforts have been made to enhance the early bone response around implants by altering the surface chemistry and/or topography of the implant. This has included biomolecular modification, electrochemical modification, alteration of surface chemistry properties, and the use of nanoparticles or nanotopographical changes.

In particular, hydrophilicity can have a substantial influence on early bone apposition to implants. For example, hydrophilic surfaces have shown increased osteogenic differentiation of mesenchymal cells through upregulation of genes associated with osteogenesis [4,5] and suppression of osteoclastogenesis [4], increased platelet activation and binding to the surface [6], downregulation of pro-inflammatory cytokines [7,8], increased expression of genes associated with angiogenesis [9], and osteoblast attachment to the implant surface [10] (for review on early wound healing responses please see [11]). Significantly greater bone-to-implant contact has been noted in the early healing period with modified SLA implants [12,13] under standard implantation conditions, but also in acute buccal dehiscence-type defects [14,15] and in circumferential defects [16,17]. Such results have been confirmed in humans with SLActive implants; a histologic and histometric study demonstrated greater osseointegration compared to SLA implants at 2 and 4 weeks [18]. Indeed, several clinical studies have demonstrated high success and survival rates, in a range of clinical situations, including use of short implants [19,20,21], early loading [22], immediate provisionalization [23], in severely resorbed mandibles [24], in the posterior maxilla and mandible [25], in the atrophic maxilla using osteotome sinus floor elevation [26], in irradiated patients [27], on long-term evaluations [28], and in general daily dental practice [29,30,31].

Recent focus has turned from hydrophilicity to the potential influence of nanostructures on implant surfaces, especially since the discovery of the presence of nanostructures on SLActive surfaces [32]. Nanostructures may have an important effect on some crucial steps in osseointegration. For example, nanotopography may play a role in modulating the inflammatory response at the implant surface [33,34], in the maturation of osteoblast-like cells through modulation of osteoblast-specific gene expression [35,36], cell differentiation [36,37], and in enhancing cell migration and growth [38]. Implants with nanostructured surfaces have also been shown to promote the activation of platelets [33] and reduce bacterial adhesion as well as promote soft tissue and bone healing.

The aim of this study was to assess any differences in surface area resulting from the presence of nanostructures on SLA titanium-zirconium surfaces (TiZr: Roxolid; Institut Straumann AG, Basel, Switzerland) and evaluate the potential effects of surface chemistry and nanotopography on the osseointegration of TiZr disks in a biomechanical implant pull-out model. Three hypotheses were evaluated in this study: (1) that mechanical pull-out forces are superior for a hydrophilic surface with nanostructures compared to a hydrophobic surface without nanostructures; (2) that mechanical pull-out forces are superior for a hydrophilic surface without nanostructures compared to a hydrophobic surface with nanostructures; (3) that osseointegration capabilities for an accelerated aged hydrophilic structure are non-inferior to those for a real-time aged hydrophilic surface.

## 2. Materials and Methods

The study was performed in accordance with the Swedish Animal Protection Law. The study was performed at the Biomedical Centre, Lund University, Lund, SE-223 62, Sweden, and ethical approval for the study was obtained from Lund University (ethical approval number M 138-14). Reporting herein follows the ARRIVE guidelines for relevant items [39].

Sixteen Swedish loop rabbits (eight male and eight female; Christer Månsson, Löberöd, Sweden) were used in this study. The animals were kept in interconnecting single cages and had ad libitum access to water and standard laboratory animal diet (RABMA^®^ (Rabbit Maintenance), 4 mm pelleted #803550, Special Diet Services, Witham, UK). Diet, room temperature and humidity were standardized. The animals were acclimatized for 1 week under test conditions prior to start of the study. A health examination was performed and only animals with no visible signs of illness were used in the study.

Five types of discs (two control and three test) were evaluated:

- Group 1: Roxolid, sandblasted, large-grit, acid-etched (Rxd SLA—Control 1)—hydrophobic, no nanostructures → dry/micro
- Group 2: Roxolid sandblasted, large-grit, acid-etched, nano structures, accelerated aged (Rxd SLA nano AA—Test 1)—hydrophobic, with nanostructures → dry/nano/AA
- Group 3: Roxolid SLActive non-aged (Rxd SLActive ‘fresh’—Control 2)—hydrophilic, without nanostructures → wet/micro
- Group 4: Roxolid SLActive, nano structures, accelerated aged (Rxd SLActive nano AA—Test 3)—hydrophilic, with nanostructures → wet/nano/AA
- Group 5: Roxolid SLActive real-time aged (Rxd SLActive RTA—Control 2)—hydrophilic, with nanostructures → wet/nano/RTA

Discs were 6.25 mm in diameter and 2 mm thick. Two discs were placed in each of the tibias for a total of 64 discs (12 for Test 1, 13 for all other groups), and were block randomized across all possible positions accounting for position and side effects. For the accelerated aged discs, the accelerated aging was done by keeping the discs at 55 °C for 21 days.

### 2.1. Pre-Surgical and Surgical Phase

A combination of intravenous ketamine (50 mg/mL, 0.35 mL/kgbw; Ketalar Vet, Pfizer AB, Solletuna, Sweden) and metetomidine (1 mg/mL, 0.15 mg/kgbw; Dormitor Vet, Orion Pharma; Espoo, Finland) was used to induce general anesthesia. A lidocaine/epinephrine solution (Xylocaine dental adrenalin) was used for local anesthesia (20 mg/mL + 12.5 mg/mL; Astra AB, Södertälja, Sweden).

The animals were anesthetized prior to surgery and the operation sites were depilated and washed with soap and ethanol. The animals were placed on their backs and covered with a sterile cover. Oxygen was provided during the entire surgery. The surgical procedure used has been previously described [40]. Briefly, in each animal, an incision was made on the proximal-anterior part of the tibia and the periosteum elevated and retained using a self-retaining retractor. Using a drill guide to ensure correct positioning, guide holes were made with a 1 mm diameter twist drill (Medartis AG, Basel, Switzerland). Two shallow platforms were then made in the bone to facilitate uniform seating of the discs by means of a custom 7.05 mm diameter bur mounted on a slow-speed dental implant drill, with copious saline solution irrigation. After the discs were seated on the platforms, polytetrafluoroethylene (PTFE) caps covering the discs to inhibit vertical bone growth and overgrowth were mounted and stabilized with a 0.25 mm titanium band, fixed using a 1.2 mm × 3 mm screw at each end. The soft tissues were then repositioned and sutured using resorbable sutures (Vicryl 4-0, FS2, Ethicon, Inc., Somerville, NJ, USA).

### 2.2. Post-Surgical Phase

Following surgery, the animals were returned to their cages, and post-operative analgesia was administered using buprenorphine (Temgesic; 0.3 mg/mL, 0.3 mL per animal; Schering-Plough AB, Stockholm, Sweden) for 3 days.

Four weeks after surgery, the animals were sacrificed using pentobarbital (Pentobarbitalnatrium, Apoteket AB, Stockholm, Sweden). The legs were removed 5 cm below the knee joint and wrapped in tissue saturated with 0.9% saline solution until analysis within 1 h of sacrifice.

### 2.3. Biomechanical Pull-Out Measurements

The procedure used for the pull-out analysis has been previously described [41]. Shortly, in each tibia sample, an incision was made through the soft tissue and the titanium band retaining the discs was exposed and removed. The PTFE caps were removed by making a hole in the center of the cap and applying pressurized air through a hollow needle. For the pull-out procedure, the tibial bone was then fixed in a specially designed device and the set-up adjusted so that the applied force was exerted at the center of each disc, perpendicular to its surface. The pull-out test was done using a Zwick Roell Z 2.5 testing machine (Zwick GmbH, Ulm, Germany) fitted with a calibrated load cell of 250 N and a cross-head speed range set to 1.0 mm/min. The load was applied until the disc was loosened and the results were recorded on a load versus time plot.

### 2.4. Statistical Analysis

The Wilcoxon signed rank test was used to compare disc types within each animal, and the Kruskal-Wallis test was used to compare the 5 groups. A multivariate mixed linear regression model was used to evaluate potential differences between the disc types, irrespective of gender, strain, side, and position. The models were adjusted for animal effect, side, disc position (proximal or distal), strain, and sex of the rabbits; animal effect was a random effect, while all others were fixed effects. The Dunnett-Hsu adjustment was used to adjust *p*-values in the case of multiple comparisons. To report non-inferiority comparison, a 90% confidence interval was used.

### 2.5. Surface Textural, Chemical, and Area Evaluation

All surface measurements were performed on discs (6.25 mm in diameter × 2 mm in height), with the appropriate surfaces applied. The same group batch was then used for both surface analyses and in vivo experimentation.

#### Static contact angle measurements

Sessile drop test with deionized water was utilized to measure the wettability of the surface of the discs (EasyDrop DSA20E, Krüss GmbH, Hamburg, Germany). Three measurements were conducted per each type of surface. Dry-stored samples were evaluated as received, whereas the samples stored in saline solution were dried by blowing a stream of argon in a laminar-flow fume hood. The size of the droplets of deionized water for the static contact angle measurements was determined to be 0.1 and 0.3 μL for wet-stored and dry-stored samples, respectively. A tangent method was used to calculate the contact angle via fitting a general conic section equation to the contour of the droplet placed on the surface of the samples.

#### Surface roughness measurements

Surface roughness at the micro-scale was evaluated using a confocal microscope (μSurf, NanoFocus AG, Oberhausen, Germany) to calculate three-dimensional roughness parameters Sa (arithmetical mean height [of the scale-limited surface], St (maximum height [of the scale-limited surface]), Ssk (skewness [of the scale-limited surface]). Three measurements each on an area of 798 × 798 μm^2^ was carried out per sample using a 20× objective, and the mean values of the calculated parameters were reported. A moving average Gaussian filter with a cut-off wavelength of 30 μm was applied.

#### X-ray photoelectron spectroscopy (XPS)

XPS analysis was performed using a PhI5000 VersaProbe spectrometer (ULVAC-PHI, Inc., Hagisono, Japan) equipped with a 180° spherical capacitor energy analyzer and a multi-channel detection system with 16 channels. Spectra were acquired at a base pressure of 5 × 10^−8^ Pa using a focused scanning monochromatic Al-Ka source (1486.6 eV) with a spot size of 200 μm and 50 W. The instrument was run in the Fixed Analyzer Transmission FAT analyzer mode with electrons emitted at 45° to the surface normal. Pass energy used for survey scans was 187.85 and 46.95 eV for detail spectra. Data were analyzed using the program CasaXPS (Version 2.3.16 www.casaxps.com). The signals were integrated following Shirley background subtraction. Sensitivity factors were calculated using ionization cross–sections corrected for attenuation, transmission-function of the instrument, and source to analyzer angle. As a result, the measured amounts are given as apparent normalized atomic concentration and the accuracy under the chosen condition is approximately ± 10%. All dry samples were removed from their packaging and directly measured. Samples stored in liquid were rinsed with ultrapure water and blow dried with nitrogen before placing the samples into the XPS chamber. Survey and detail spectra of O 1s, Ti 2p, N 1s, C 1s (including K 2p and Mg KLL), Si 2p, S 2p, Na KLL, P 2p (including Zn 3s and Pb 4f), Al 2p, F 1s, Zr 3d, Al 2s and Ca 2p (including Zr 3p1/2), Ca LMM, Mg 1s and Cl 2p were measured on each sample.

#### Scanning electron microscopy (SEM)

The samples were then analyzed using an scanning electron microscope (Zeiss Supra 55, QS Nr. 57113, Oberkochen, Germany) with a field electron emitter (FE-SEM). SEM is reported at 500× and 20,000× in the present study.

#### Surface area analysis

For the in vitro surface area analysis, Roxolid discs were prepared with a modified-SLA-like surface (mod-dry/micro) and a SLActive surface, accelerated aged and then air dried under a laminar flow hood (dry/nano). Atomic force microscopy (AFM) is the most suitable technique for measuring nanoscale surface topography of hard surfaces, but the sandblasting process leads to undesirable artefacts in the measurement process; the sandblasting step was therefore omitted without impacting the low-micro and nanotopography, which are a result of the etching and aging process.

AFM measurements were performed using a JPK Nanowizard 4 (Bruker Nano GmbH, Berlin, Germany) with a direct drive cantilever holder and SSS-NCHR cantilevers with a tip radius of 2–3 nm (Nanotools GmbH, Munich, Germany) in dynamic mode. An area of 2 × 2 µm^2^ was scanned for every measurement at 0.25 Hz/line and recorded using 2048 × 2048 points. The data were analyzed using µSoft Analysis XT software (version 5.1.1.5944; NanoFocus AG, Oberhausen, Germany) according to ISO 25,178 using a Gaussian filter with a cut-off wavelength of 0.25 µm and the area ratio between mod-dry/micro and dry/nano was estimated as (SdrmodMA/100 + 1)/(SdrMA/100 + 1), where Sdr is the mean developed interfacial area ratio. For each sample group, 5 discs were measured at two random positions.

## 3. Results

One animal from Lot 1 developed an infection during the healing phase, unrelated to the implanted materials, which was unable to be successfully treated. This animal was euthanized to avoid suffering, and the implanted discs were not evaluated; i.e., the pull-out force of a total of 60 implants was evaluated and reported.

### 3.1. Surface Properties Characterization

The different surfaces were characterized for surface morphology, roughness, chemistry, wettability, and surface area.

#### 3.1.1. Surface Morphology, Roughness, and Chemistry

##### Surface morphology:

**Figure 1:**
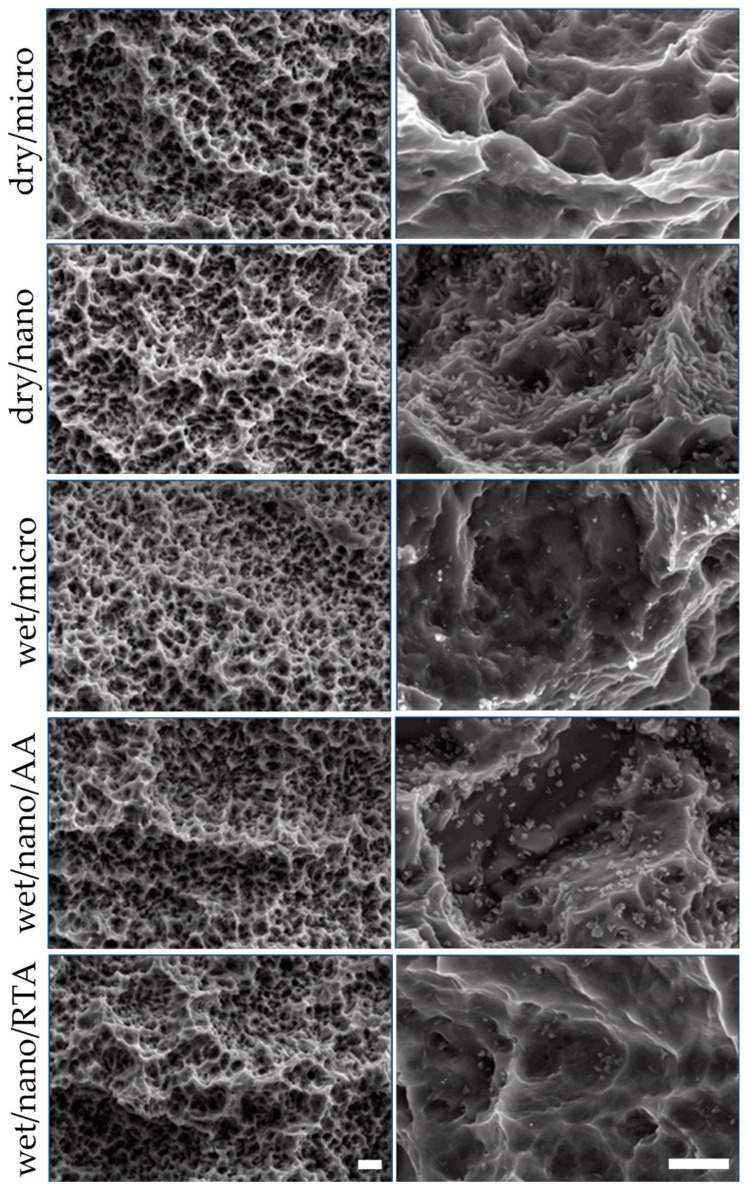
SEM images demonstrating comparative surface morphologies of micro- versus nano-structured materials. Left column: Low magnification showing macro-roughness; Scale bar = 4µm; Right column: High magnification showing micro- and nano-roughness; Scale bar = 400 nm.

As characterized by SEM, the surface morphology analysis demonstrated that the dry/micro and wet/micro surfaces were micro-textured and absent of any nanostructures. The other three groups (dry/nano, wet/nano/AA and wet/nano/RTA) demonstrated high density coverage of nanostructures. Amongst the wet/nano/AA and wet/nano/RTA groups, the AA showed a clearly higher density of visible nanostructures, as compared to the RTA group (Figure 1).

##### Surface roughness

All five groups featured nearly identical Sa, St, and Ssk values, demonstrating that the micro-roughness was consistent across all the production steps for the group generations. It should be noted that any nanofeatures would not be captured on such a measure as they are below the level of detection (Table 1).

##### Surface chemistry

XPS data of the samples analyzed revealed that the highest carbon content was present on the surface of dry/micro sample, originating from the contamination of the samples through hydrocarbon-based species from the ambient atmosphere. All wet samples contained substantially lower amounts of carbon on their surface, having been packed in nitrogen atmosphere directly following the micro-structuration process and then transferred and stored in saline solution until analysis. The nitrogen atmosphere packaging and saline storage steps appear to have protected them from carbon contamination (Table 2). No unexpected elements were detected.

##### Wettability

Both dry/micro and dry/nano groups featured a hydrophobic surface, with static contact angles (SCA) of 123.0° ± 0.3° and 100.3° ± 3.1°, respectively. The latter exhibited a slightly less hydrophobic surface. The remaining three wet groups (i.e., wet/micro, wet/nano/AA, and wet/nano/RTA) all showed a comparative super-hydrophilic surface with a SCA of 0°.

#### 3.1.2. Surface Area Evaluation

**Figure 2:**
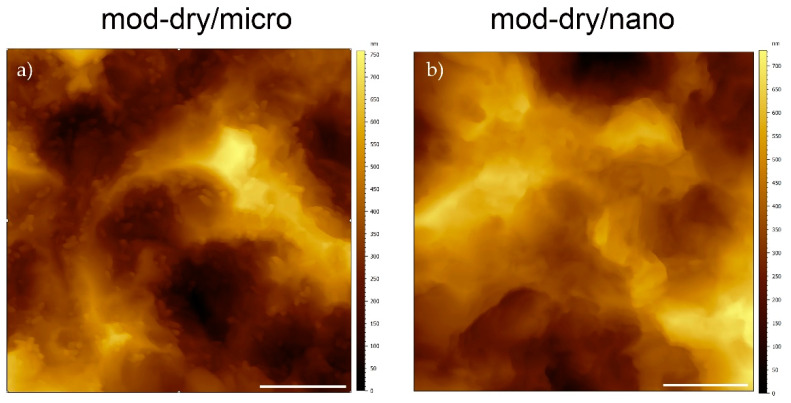
AFM topography images of nano-structured (**a**) and micro-structured (**b**) implants.

AFM analysis showed that Sdr of the scale-limited surface for dry/nano (Sdrdry/nano; representing the nanotopography of SLActive) was 183 ± 36%, significantly greater than the Sdr for the mod-dry/micro surface (Sdrmod-dry/micro; representing the nanotopography of SLA) of 72 ± 36% (*p* < 0.001), indicating that the Roxolid dry/nano surface area is 64% greater than that of the Roxolid mod-dry/micro surface due to the presence of nanostructures. Nanostructures, and the subsequent increase in surface area, were clearly visible in the AFM topography images (Figure 2).

### 3.2. Biomechanical Pull-Out Measurements

**Figure 3:**
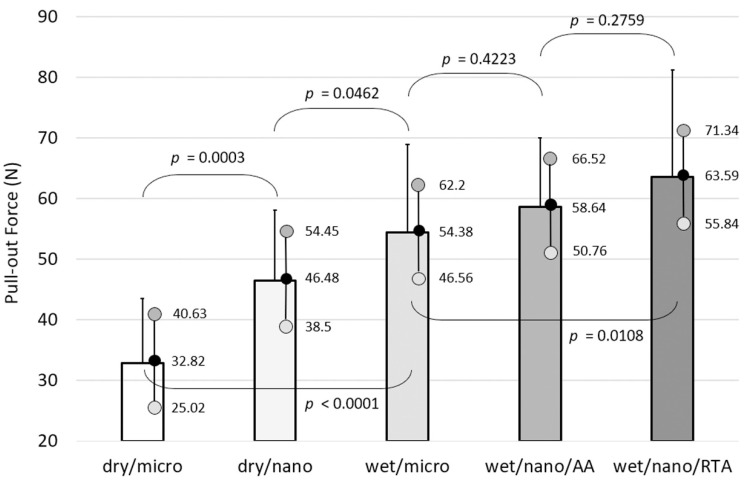
Association between pull-out force and treatment type (implant type), adjusted by gender, strain, side, and implant position. Bars represent adjusted mean values with standard deviations. Black dot = adjusted mean with real value; dark grey w/black outline dot = upper 95% confidence interval limit with real value; light grey w/black outline dot = lower 95% confidence interval limit with real value. *p* values are provided; *p* ≤ 0.05 is considered significant.

The highest mean pull-out force was recorded for the wet/nano/RTA implants (64.5 ± 17.7 N). The dry/micro implants showed the lowest mean pull-out force (33.8 ± 10.7 N). Pull-out forces were also greater for dry/nano/AA implants (56.3 ± 16.2 N), wet/micro implants (59.0 ± 15.2 N), and wet/nano/RTA implants (64.5 ± 17.7 N), when stratified by surface topography, surface chemistry, and aging procedure. There were no significant differences within the groups with respect to implantation side or gender. Using a multivariable mixed linear model, all disc types demonstrated significantly greater pull-out force than the dry/micro discs. Dry/nano/AA implants showed significantly lower pull-out forces compared to the hydrophobic groups. Pull-out forces for the wet/micro implants were lower than those for the wet/nano/AA group and significantly lower than those for the wet/nano/RTA group. The association between pull-out force and treatment type (i.e., implant type) demonstrates greater pull-out forces with nanostructured and hydrophilic surfaces, with the wet/nano/RTA implants having the largest pull-out force (Figure 3).

**Figure 4:**
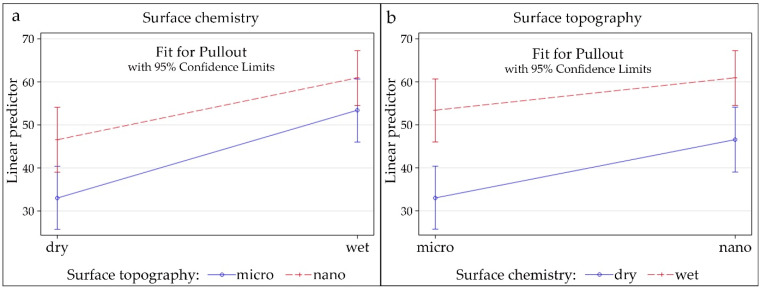
(**a**) Simple effects of surface chemistry; (**b**) Simple effects of surface topography.

To determine the comparative degree of contribution of the two key surface parameters (surface topography or wettability) to osseointegration, a multivariable linear mixed model was performed that incorporated surface wettability, surface topography, interaction between wettability and topography, gender, strain side, and position as random effects (Table S1). The analysis showed that wettability and surface topography both have a significant effect (*p* < 0.0001 for both), but the effect of the interaction between wettability and topography is not significant (*p* = 0.1056). Of the other factors, only position showed a significant effect (*p* < 0.0001); all others (gender, strain and side) were not significant. The combinations of both factors, i.e., the simple effects, are shown in Figure 4a (for surface chemistry) and Figure 4b (for surface topography). Pull-out forces were greater for hydrophilic than hydrophobic surfaces, for both types of topography (micro and nano), but higher for nano (Figure 4a; surface chemistry had the highest effect (*p* < 0.0001 for both surface topographies). Similarly, pull-out forces were greater for nano-surface topography than for micro, but higher for hydrophilic than hydrophobic surfaces (Figure 4b); the effect is greater for hydrophobic surfaces (*p* = 0.0009) than for hydrophobic surfaces (*p* = 0.0213).

Non-inferiority of the accelerated versus the real-time aged discs was evaluated using the average effect and its two-tailed 90% confidence interval (equivalent to a one-tailed 95% confidence interval using the previously described regression model (Table S2). There was a non-significant difference between the pull-out force for the accelerated aged discs and the real-time aged discs (*p* = 0.1783); the mean pull-out force of the accelerated aged discs was 5.06 N lower. A tolerance range of 11.44 N would be necessary to indicate that the pull-out force of the accelerated aged surface is at least as good as that of the real-time aged surface.

## 4. Discussion

This study was designed to evaluate the influence of surface chemistry and surface nanotopography on osseointegration. The extent of osseointegration, as measured by the pull-out forces necessary to remove them in a biomechanical model, was assessed between (1) hydrophilic surfaces with nanostructures versus hydrophobic surfaces without nanostructures; (2) hydrophilic surfaces without nanostructures versus hydrophobic surfaces with nanostructures; and (3) accelerated aged versus real-time aged surfaces.

For the first hypothesis, it was confirmed that superior osseointegration was achieved with an SLActive (hydrophilic), nanostructured surface compared to an SLA (hydrophobic) surface without nanostructures. Furthermore, all of the evaluated groups showed superior pull-out properties over the SLA surface, indicating that osseointegration capabilities are significantly enhanced by both chemistry (hydrophilicity) and topography (nanostructures).

In the second hypothesis, the hydrophilic surface without nanostructures was confirmed to have greater pull-out properties than the hydrophobic surface with nanostructures. This indicated that surface chemistry may have a greater effect on osseointegration than nanotopography, although both were found to have a significant effect (*p* < 0.0001 for both). The effect of the interaction between chemistry and topography, however, was not significant (*p* = 0.1056), suggesting that they contribute independently. Pull-out values were significantly higher for hydrophilic/nanostructured surfaces compared to hydrophobic/nanostructured surfaces (*p* < 0.0001), suggesting that osseointegration is enhanced by hydrophilicity on both types of surface topography. Similarly, the effect of nanotopography was significantly greater than the effect of microtopography (adjusted *p*-values of 0.0009 and 0.0213 for hydrophobic and hydrophilic surfaces, respectively). This indicates that, although surface chemistry has a significantly greater effect than topography, the effect of topography seems to be stronger on hydrophobic compared to hydrophilic surfaces. This is confirmed by Figure 4, where the slopes are steeper for the effects of surface chemistry (Figure 4a) than the effects of surface topography (Figure 4b), indicating that the difference between hydrophilic and hydrophobic is higher for both topographies than the difference between microtopography and nanotopography for both types of surface chemistry. In addition, the effect of surface topography was more pronounced on hydrophobic surfaces. Finally, for the third hypothesis, it was confirmed that the pull-out forces with the accelerated aged hydrophilic surface are non-inferior to those for the real-time aged hydrophilic surface.

Confirmation of the first hypothesis is in line with previous studies where superior osseointegration was obtained with a combination of hydrophilicity and nanostructures. For example, in a similar biomechanical study in rabbits, SLA discs with and without nanostructures, and modified SLA (hydrophilic) discs with low- or high-density nanostructures were evaluated [42]. The pull-out forces indicated that the combination of hydrophilicity and a nanostructured surface resulted in the greatest bone response. Results from in vivo studies have shown greater bone apposition at the modified (hydrophilic) compared to the standard SLA surface. For example, Bornstein and colleagues evaluated modified SLA and standard SLA implants in the mandibles of dogs, and showed that significantly more bone was formed in contact with the modified SLA implants [12]. Histological and histometric examination of hydrophilic (SLActive) compared to hydrophobic (SLA) implants in humans, where implants were placed and retrieved after 7, 14, 28, and 42 days, also indicated more newly formed bone at the SLActive surface in early healing, after 2 and 4 weeks [18].

The focus on nanotopography in recent years has suggested that the presence of nanostructures on implant surfaces, regardless of the surface chemistry properties, may be the most important factor in enhancing osseointegration, particularly in the early bone response phase. Liddell and colleagues examined the effects of nanocrystal deposition, ultraviolet light, or sodium lactate treatment on titanium implants placed in the femora of rats, with pull-out testing performed after 5, 9, or 14 days [43]. Greater pull-out forces were found with the nanocrystalline deposit implants at all time points, and the authors suggested that nanotopography had a more profound effect on bone anchorage [43,44] also suggested that cell spreading and protein expression were more substantially regulated by changes in titanium surface topography than wettability (i.e., hydrophilicity), and suggested that the influence of wettability was more apparent on smooth than rough surfaces [44]. Others have shown that nanotopography and hydrophilicity both have an influence on osteoblast cell response, such as Gittens and colleagues, who indicated that osteoblast-like cell maturation and local factor production was synergistically influenced by nanostructures on the implant surface, without any exogeneous soluble factors [35]. In contrast, without the introduction of nanostructures, the surfaces suppressed differentiation of osteoblasts and local factor production. The authors did suggest, however, that surface wettability may be partly responsible. In our study, the results indicated that surface chemistry alterations had a greater influence overall than alterations of the surface topography. One of the few previous studies that has also suggested this looked at the osteogenic response to hydrophilic and nanostructured titanium surfaces and found that stimulation of human mesenchymal stem cells and human osteoblasts was more affected by hydrophilicity than by the presence of nanostructures [45]. The significant increase in surface area conferred by nanostructures, as demonstrated by the AFM analysis performed as part of this study, may also play a role in the greater expression of cell and protein factors.

Confirmation of the third hypothesis has direct implications on the implant production process. Under normal storage conditions, nanostructure formation on Roxolid materials proceeds significantly slower than those formed on Titanium grade 4 materials (roughly 6 versus 1 month/s, respectively, to reach the plateau at which spontaneous nanostructure evolution ceases). Implants are thus stored accordingly, with Roxolid implants obliged to be stored at least six months prior to shipping. With the present study, it is now confirmed that the nanostructures formed in an accelerated-aged manner (roughly three weeks to reach the aforementioned plateau) result in comparable levels of osseointegration than that which is found on real time-aged nanostructures. The knock-on effect is thus on the storage time of the implants, with final-packaged Roxolid-based implants able to be shipped at least three weeks after production, similar to titanium grade 4 implants.

The surface analysis resulted in several interesting observations. Amongst the wet/nano/AA and wet/nano/RTA groups, the AA process appears to have promoted the growth rate of these nanostructures, resulting in a higher density of nanostructures visible on the AA group as compared to the RTA. It is unknown if simply leaving the RTA group longer in the saline solution for a longer period of time would have resulted in a higher density of nanostructures, or if the nanostructure formation at room temperature simply reaches a plateau whereupon nanostructure formation ceases to progress. For the surface roughness measurements, even though this study concentrated on nanotopography, it was important to demonstrate that the underlying micro-topography was consistent. This consistency permitted to isolate the impact of the nanotopography in terms of osseointegration as the micro-topographical parameter could be considered fixed. For the XPS measurements, even though the base material was consistent across the groups, the apparent relative titanium surface amount was detected to be lower in the dry groups. As this is a relative percentage of atoms on the surface, the higher percentage of carbon atoms on the dry group surfaces artificially drove down the titanium percentage. The types of bonds pertaining to the carbon atoms can also be determined in the XPS measurement. Most of the carbon bonds were found to be that of carbon-carbon, indicative of hydrocarbon presence, thus supporting the presence of a carbon contaminating layer on the surface of the dry implants. The lower amount of carbon contaminating layer on the surface of the dry/nano as compared to dry/micro can be explained by the fact that production of this group goes through a saline solution intermediate step, which prevents hydrocarbon buildup. However, once removed from the saline and dried, a hydrocarbon layer can then start to form.

Our results demonstrate that alteration of the surface chemistry and topography of TiZr implants can have a profound influence on the degree of osseointegration potential. We believe that the results obtained in our investigation add to the body of knowledge, especially in the demonstration of the relative influence of hydrophilicity and nanotopography. As mentioned above, there are numerous examples in the literature of the benefits of a hydrophilic implant surface in the increased gene expression and production of factors involved in osseointegration, and in the enhanced response and proliferation of osteoblastic cells. This may be in part due to the presence of nanotopography on hydrophilic surfaces [42]. However, we also demonstrate that the presence of nanostructures on hydrophobic surfaces can also significantly increase the capacity for osseointegration at the implant surface. This may help in the continuing drive to improve and refine dental implant surfaces to make osseointegration faster, therefore in turn potentially leading to more rapid treatment options for patients.

In conclusion, the results of our investigation clearly show that the introduction of hydrophilicity to the TiZr implant surface significantly increases the capability for osseointegration, measured by the pull-out force evaluation, both on surfaces with and without nanostructured topography. We also show that the introduction of nanostructures on the surface of the implants significantly increases the capacity for osseointegration, as measured by the pull-out forces, and that accelerated aging of the implants is non-inferior for osseointegration to real-time aging.

## Figures and Tables

**Table 1:** Surface roughness measures.

Sample	Sa (µm)	Sa (SD)	St (µm)	St (SD)	Ssk	Ssk (SD)
dry/micro	1.35	0.04	8.89	0.32	0.37	0.02
dry/nano	1.32	0.06	8.64	0.44	0.38	0.03
wet/micro	1.33	0.06	8.95	0.38	0.40	0.03
wet/nano/AA	1.31	0.07	8.93	0.29	0.38	0.03
wet/nano/RTA	1.35	0.04	8.90	0.21	0.40	0.03

**Table 2:** Apparent normalized atomic concentration of detected elements as measured by XPS.

Sample	O (At. %)	Ti (At. %)	C (At. %)	Zr (At. %)
SLA	38.1 ± 3.03	12.9 ± 0.97	46.6 ± 4.24	2.4 ± 0.26
SLAnano AA	51.0 ± 1.93	16.9 ± 1.61	27.7 ± 3.22	4.4 ± 0.33
SLActive Fresh	58.2 ± 0.89	21.9 ± 0.54	16.3 ± 2.08	3.6 ± 0.71
SLActive Nano AA	58.3 ± 0.19	20.5 ± 0.52	16.6 ± 0.37	4.6 ± 0.36
SLActive Nano RTA	56.8 ± 2.79	20.5 ± 1.20	18.3 ± 4.19	4.4 ± 0.20

## Data Availability

The data presented in this study are available on reasonable request from the corresponding author. The data are not publicly available due to the proprietary nature of the thermal aging process applied to the groups in this study.

## Supplementary Materials

The following are available online at https://www.mdpi.com/article/10.3390/ma14071678/s1, Table S1: Multivariate mixed linear regression model: Association of pull-out force and surface characteristics, adjusted for other factors (gender, strain, position, side). Table S2: Non-inferiority comparison of accelerated and real-time aged discs (hydrophobic with nanostructures); aging procedure effect on pull-out force adjusted by side, position, strain, gender, and other animal effects.
