# Supplementary material for: Greater Osseointegration Potential with Nanostructured Surfaces on TiZr: Accelerated vs. Real-Time Ageing
Source: Materials (Basel). 2021 Mar 29;14(7):1678. doi: 10.3390/ma14071678 (PMC8036800; doi:10.3390/ma14071678)
Supplement: Supplementary file 1 [file materials-14-01678-s001.pdf]

materials

Supplementary Materials

Table 1. Multivariate mixed linear regression model: Association of pull-out force and surface characteristics, adjusted for other factors (gender, strain, position, side.

Factor	Value	Outcome: Pull Out Force [N]						
		Regression estimate	SE	p-value	Adjusted mean	95% CI for the adjusted mean	p-value*	p-value (for the overall effect of a factor)
Surface Chemistry	Hydrophobic	-14.820	2.720	<.0001	39.635	32.381 to 46.889	<.0001	<.0001
	Hydrophilic	0			57.842	50.774 to 64.911		
Surface Topography	Micro	-6.849	2.619	0.0125	43.620	36.4207 to 50.820	<.0001	<.0001
	Nano	0			53.857	46.737 to 60.977		
Interaction of Surface Chemistry and Surface Topography	Hydrophobic Micro	-6.775	4.092	0.1056				0.1056
	Hydrophobic Nano	0						
	Hydrophilic Micro	0						
	Hydrophilic Nano	0						
Gender	Female	-5.010	9.377	0.5961	46.234	33.096 to 59.371	0.5961	0.5961
	Male	0			51.244	41.196 to 61.291		
Strain	Black	0.836	9.926	0.9333	49.157	34.848 to 63.465	0.9333	0.9333
	White	0			48.321	38.811 to 57.83		
Position	Distal	8.070	1.884	0.0001	52.774	45.666 to 59.882	0.0001	0.0001
	Proximal	0			44.704	37.584 to 51.823		
Side	Left	-2.941	1.879	0.1253	47.268	40.158 to 54.378	0.1253	0.1253
	Right	0			50.209	43.095 to 57.324		

Table 2. Non-inferiority comparison of accelerated and real-time aged discs (hydrophobic with nanostructures); aging procedure effect on pull-out force adjusted by side, position, strain, gen-der, and other animal effects.

Factor	Value	Outcome: Pull Out Force [N]						
		Regression estimate	SE	Adjusted mean	95% CI for the adjusted mean	p-Value*	Average effect of the factor	(90% CI)§
Intercept		61.928	11.791					
Group	Roxolid SLActive nano AA	-5.060	3.429	58.159	47.166 to 69.151	0.1783	-5.060	-11.437 - 1.317
	Roxolid SLActive RTA	0		63.219	53.302 to 73.136			
Gender	Female	-3.5361	11.379	58.921	40.641 to 77.200			
	Male	0		62.457	48.385 to 76.529	0.7639		
Strain	Black	-1.3861	12.205	60.000	39.702 to 80.289	0.9124		
	White	0		61.382	48.262 to 74.501			
Position	Distal	12.3141	6.107	66.846	53.969 to 79.723	0.0785		
	Proximal	0		54.532	43.520 to 65.543			
Side	Left	-4.810	3.42972	58.284	47.292 to 69.276	0.1983		
	Right	0		63.094	53.177 to 73.011			
